# Two-Step Extraction of the Lower First Molar for Class III Treatment in Adult Patient

**DOI:** 10.1155/2016/1580313

**Published:** 2016-09-06

**Authors:** Kélei Cristina de Mathias Almeida, Ricardo Fabris Paulin, Taísa Barnabé Raveli, Dirceu Barnabé Raveli, Ary Santos-Pinto

**Affiliations:** ^1^Department of Orthodontics and Pediatric Dentistry, Araraquara School of Dentistry, Universidade Estadual Paulista (UNESP), Rua Humaitá 1680, 14801-903 Araraquara, SP, Brazil; ^2^Discipline de Occlusion and Orthodontics, Paulista University (UNIP), SGAS 913, Asa Sul, 71918-000 Brasília, DF, Brazil

## Abstract

The aim of this article is to describe a case report of Class III malocclusion treatment with lower first molar extraction. The 27-year-old Caucasian male patient presented a symmetric face with a straight profile, hyperdivergent growth pattern, molar and cuspid Class III relation, and an anterior crossbite as well as a mild crowding on cuspids area, in both upper and lower arches and a tendency to posterior crossbite. The treatment was performed by the use of Haas expansion appliance followed by an initial alignment and leveling of the upper and lower arches with a fixed edgewise appliance, extraction of lower teeth aiming the correction of the incisors proclination and end the treatment with a Class I molar relationship. It resulted in a significant change in the patient's profile, dentoalveolar Class III correction, upper arch expansion, leveling and alignment of the upper and lower arches, and improvement of tipping of the upper and lowers incisors. In cases of a dentoalveolar compensation in well positioned bone bases the treatment with fixed appliances is an alternative and extraction of lower teeth is considered.

## 1. Introduction

Treatment for Class III malocclusion is still challenging for orthodontists and moreover for adult patients that present skeletal concern and wish conservative approach [1]. Fortunately, the malocclusion Class III represents a small percentage of all malocclusions among orthodontic patients [2]. The prevalence of this malocclusion was reported between 1% [3, 4] and 10% [2], depending on ethnic origin [4, 5], gender [1, 6], and age [7], as well as the diagnostic criteria [8].

For adult patients with this malocclusion, there are two main treatment options: dental compensation and orthognathic surgery [1, 9–13]. The value of skeletal discrepancies usually determines whether a surgical correction is appropriate. However, in borderline cases, a balance of soft profile will help determine whether patients can deal with dental compensation [14].

A treatment option for patients that are reluctant to undergo surgery or that are satisfied with their facial appearance is dentoalveolar compensation without skeletal correction [1]. In general, fixed appliances with extractions are considered the best option for adult Class III nonsurgical treatment [14].

Traditionally, the extraction of four premolars (first lower and upper seconds) is the most common option; however, other choices extractions can also be used [15–17]. When lower third molars are present, the extraction of lower first molars can be considered for Class III treatment as long as they present one or more of the following conditions: root canal treatment, large cavities, severe periodontal disease, and extensive restoration [18].

This treatment option corrects the molar sagittal position, providing a Class I relationship between upper first molars and lower second and mesial placement of these teeth can facilitate anticlockwise mandibular rotation. However, this treatment approach is not indicated for all patients for it requires an additional orthodontic biomechanics to manage first molar space closure which takes very long. Also, lower second molars tend to tip mesially and lingually.

The aim of this paper is to present a case report in which a Class III malocclusion was treated with first lower molars extraction and complete fixed appliances.

## 2. Clinical Case

**Figure 1:**
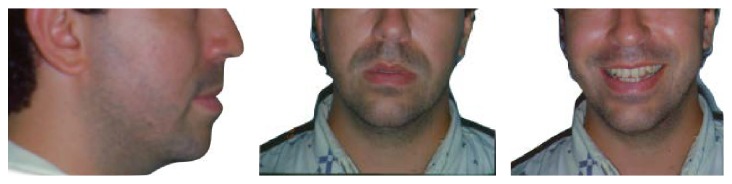
Patient with symmetric face with a convex profile and a hyperdivergent growth pattern.

**Figure 2:**
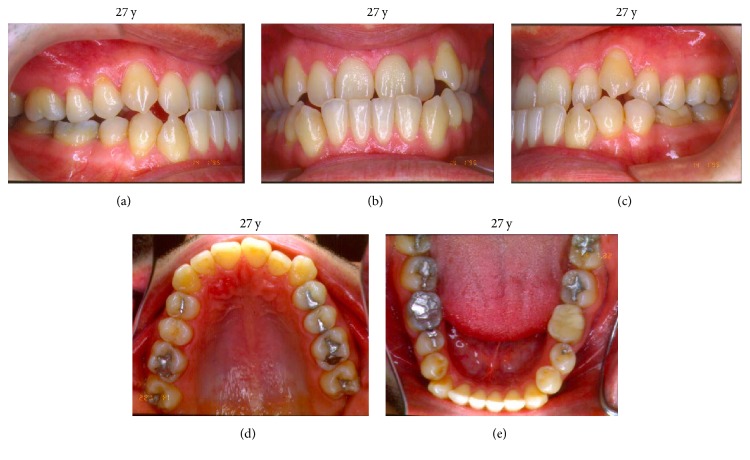
Intraoral exam: (a) molar and cuspid Class III relation; (b) anterior crossbite; (c) tendency to posterior crossbite; (d, e) mild crowding on cuspids area in both upper and lower arches.

During clinical examination, it was observed that the 27-year-old caucasian male patient presented a symmetric face with a convex profile and a hyperdivergent growth pattern (Figure 1). The intraoral exam revealed a molar and cuspid Class III relation and an anterior crossbite as well as a mild crowding on cuspids area in both upper and lower arches and a tendency to posterior crossbite (Figure 2).

**Figure 3:**
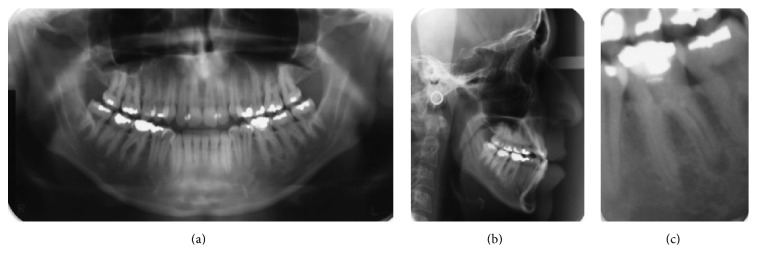
(a) Panoramic X-ray showed integrity of the permanent teeth and absence of upper third molars; (b) cephalometric analysis showed a severe vertical pattern, convex profile, increased lower facial height, a slightly retruded maxilla in relation to the cranial base, and a well-positioned mandible. The upper incisors were in a good inclination while the lower incisors were uprighted; (c) endodontic lesion in tooth 36.

Panoramic X-ray showed integrity of the permanent teeth and absence of upper third molars although the lower third molars were present and already erupted into the arch (Figure 3(a)). The cephalometric analysis showed a severe vertical pattern, convex profile, increased lower facial height, a slightly retruded maxilla in relation to the cranial base, and a well-positioned mandible (Figure 3(b)). The upper incisors were in a good inclination while the lower incisors were uprighted. The cephalometric data are showed in Table 1.

According to cephalometric analysis, the diagnosis was dentoalveolar Class III with anterior crossbite. The initial treatment plan consisted of palatal expansion followed by fixed appliances on both arches, with a probable extraction of lower teeth.

**Figure 4:**
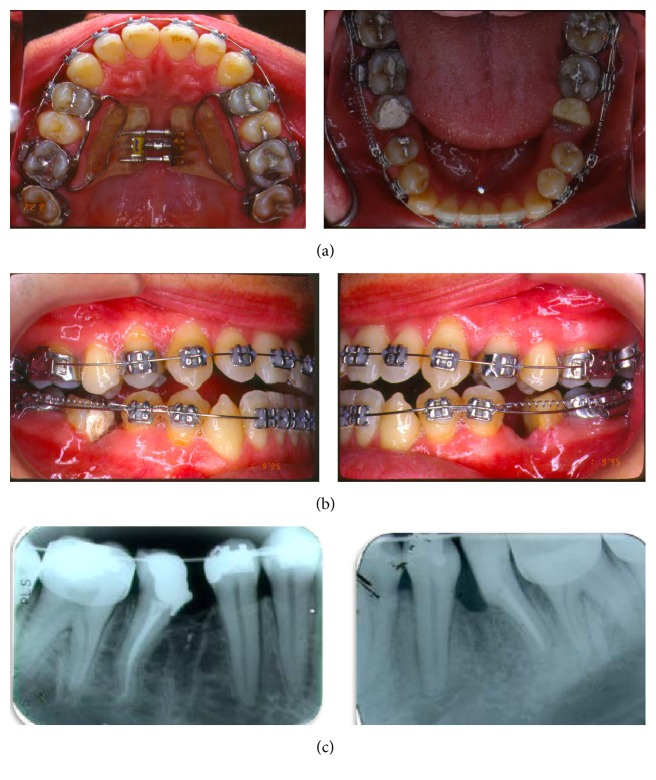
(a) The treatment was conducted initially by the use of Haas expansion appliance; (b, c) the extraction was performed in two steps, first with the extraction of the mesial root of the first molar, followed by the extraction of the distal root after closing the resultant space with a retraction loop.

The treatment was conducted initially by the use of Haas expansion appliance (Figure 4(a)) followed by an initial alignment and leveling of the upper and lower arches with a fixed edgewise appliance. The aim of using the Haas appliance was just a dentoalveolar expansion as the patient was an adult so there was no possibility of opening the midpalatal suture. A 5/16 Class III elastic with 8 ounces was used associated with a lip bumper in order to achieve maxilla dentoalveolar protraction.

**Figure 5:**
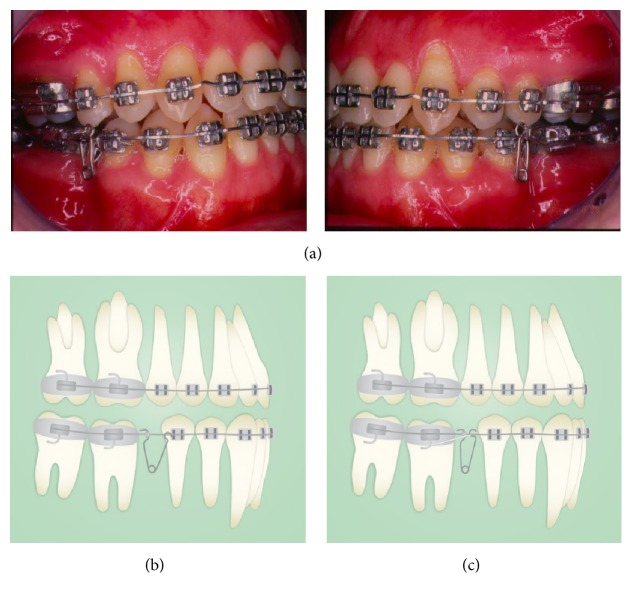
After the alignment of the lower anterior segment, an H loop was inserted to finish the space closure by second molars avoiding its tipping. Class III elastics were used during this phase of the treatment.

In order to correct the anterior crossbite, the treatment plan would include the extraction of lower teeth. So it was decided for the extraction of the lower first molars, as the lower third molars were present while there were no upper third molars, aiming at the correction of the incisors proclination and end the treatment with a Class I molar relationship. The extraction was performed in two steps, first with the extraction of the mesial root of the first molar (Figures 4(b) and 4(c)) followed by the extraction of the distal root after closing the resultant space with a retraction loop. These two steps of extraction were done to avoid anchorage loss and tipping of second molars in the first moment when premolars were retracted to open space for canine alignment. For that purpose, it was used a closed NiTi coil spring (250 gr) applied from the second molar to the second premolar, which was tied together to the first premolar. After the alignment of the lower anterior segment, an H loop (Figure 5) was inserted in order to finish the space closure by second molars mesial movement avoiding its tipping. Class III elastics were used during this phase of the treatment.

**Figure 6:**
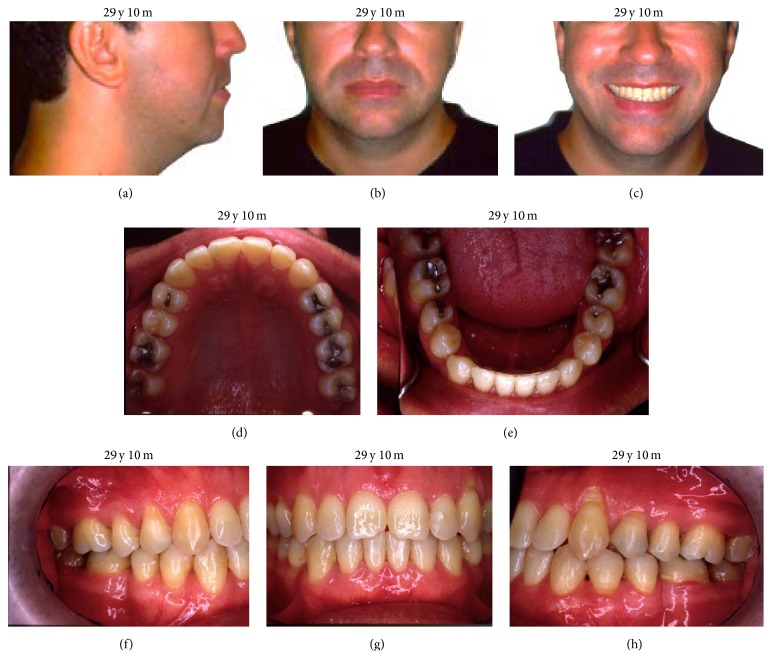
(a, b, c) Alteration in the patient's profile after treatment, dentoalveolar Class III correction, upper arch expansion, and leveling and alignment of the upper and lower arches; (d, e, f, g, h) intraoral pictures after complete treatment.

**Figure 7:**
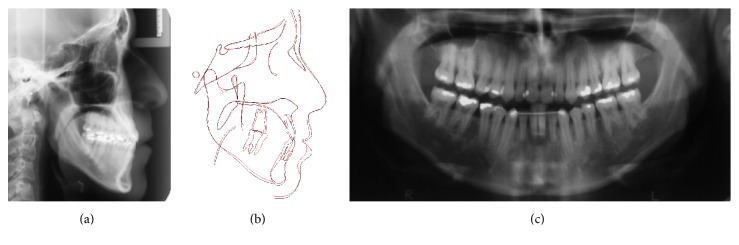
(a, b) Movements of the upper and lower incisors can be noted in the cephalometric X-ray; (c) Upper removable appliance and a 3-3 lower retainer used for retention.

The treatment resulted in a significant alteration in the patient's profile (Figures 6(a)–6(c)), dentoalveolar Class III correction, upper arch expansion, and leveling and alignment of the upper and lower arches (Figures 6(d)–6(h)). A slight protrusion was also observed and lingual tipping of the upper and slight retraction and lingual tipping lower incisors dental compensation for unfavorable skeletal discrepancy the final lateral radiograph analysis (Figures 7(a) and 7(b)) are presented in Table 1. An upper removable appliance and a 3-3 lower retainer were used for retention (Figure 7(c)).

**Figure 8:**
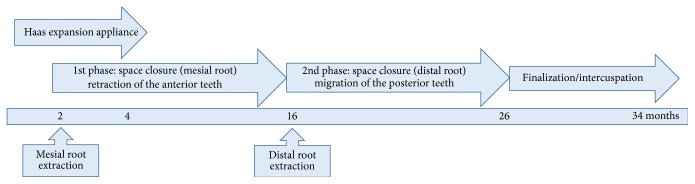
Flowchart of treatment with Haas expansion appliance.

Figure 8 summarizes the treatment with Hass expansion appliance that was carried out as 1 turn per day (0.2 mm) during 2 weeks and the appliance was kept in retention for 4 months. Mesial root extraction was performed after 2 months of treatment. The first phase of the space closure occurred at 12 months with retraction of the premolars to align anterior teeth. The distal root extraction was performed after 14 months of treatment. The second phase of the space closure occurred at 10 months with molars mesialization. Finalization and intercuspidation occurred during 8 months. Total treatment time was 34 months.

## 3. Discussion

When Class III malocclusion treatment starts up after the facial growth, the options are more limited; usually tooth extraction associated with orthodontic mechanics is considered the only option for nonsurgical treatment [14].

Usually the extraction of the four premolars would be the first choice, but the first molars may be an option when they have endodontic treatment, large cavities of caries, severe periodontal involvement, and extensive restorations. Other situations in which first molars can be extracted are hypoplastic lesion, apical pathology, and lower anterior teeth crowding [18].

This case was conducted with extraction of the first molars because they presented extensive restorations and the lower left first molar (36) showed a periapical lesion in the distal root. It was also considered the absence of up third molars, invigorating this diagnosis. Among the advantages of opting for such conduct, it is the Class I dental relationship (molar and canine), established by the second molars, but among the disadvantages, there are more complicated orthodontic mechanics and prolonged duration of treatment [19].

The upper dental crowding was treated with the rapid expansion of the arc with Haas appliance (Figure 4). This therapy promoted the increase of the arch perimeter with vestibular inclination of posterior teeth without moving the anterior teeth to this sense. In adults, increased skeletal transverse dimension of the palate promoted by ERM is small [20, 21], being predominantly dentoalveolar. In this case, changing the positioning of the maxillary bone may have occurred, most likely due to an “alveolar bone remodeling,” resulting sagittal change of point A, changeable with orthodontic therapy [22].

The cephalometric superimposition (Figures 7(a) and 7(b)) shows that the molar inclined to mesial position, but this movement was controlled by segment extraction of the first molars roots. The extraction of the first molars mesial root allowed the alignment and correction of overjet, while the extraction of the distal roots allowed establishing a class I relationship of canines and the remaining space enclosed by mesialization of the second molar with spring H support. The disadvantage of this procedure was the need for endodontic treatment of these teeth.

Changes in positions of lower incisors were significant, where there was a lingualization, extrusion, and retraction with alveolar remodeling. Also, the mesial movements of the molars produced a mild anticlockwise rotation of the plans occlusal and mandibular [16].

As only the first lower molars were extracted, avoiding significant changes in the upper lip, it would be undesirable for the facial profile of this patient. Labial movement of the upper incisors was achieved by the use of the Class III elastics during all phases of treatment.

After the end of orthodontic treatment, in the extraction sites, it is common to find interdental gingival crevicular [14, 23]. These are defined as mesial and distal tissue invagination having a depth of, at least, 1 mm [24]. The presence of these crevices may have clinical implications, not only in terms of orthodontic recurrence but also to maintain the periodontal health. Thus, performing surgery to prevent opening interdental space and maintain periodontal health is shown [25, 26].

In the case described here, there was no sagital skeletal component, although the patient presented an increased lower facial height. As there was no intention of a surgical approach, an extraction therapy was considered a viable option.

The treatment was successfully conducted. There was the correction of the anterior crossbite, tendency of posterior crossbite, and molar/cuspid relationship. The enhancement of the facial profile was also observed.

## 4. Conclusion

The Class III treatment in adult patients is very complicated and often requires surgical correction. But it is known that in some situations instead of a skeletal dysplasia there is a dentoalveolar compensation in well positioned bone bases. In these cases the treatment with fixed appliances is an alternative, and in some instances, extraction of lower teeth is considered.

## Figures and Tables

**Table 1:** Cephalometric analysis.

Cephalometric data	Standard values of Caucasian	Pretreatment	Posttreatment
*SNA*	82° ± 2°	79.0°	81.0°
*SNB*	80° ± 2°	79.0°	79.0°
*ANB*	2° ± 2°	0°	2.0°
*SND*	76°/77°	77.0°	77.0°
*Mand. plane*	32°	42.0°	39.0°
*SNOP*	14°	15.4°	12.6°

*1.NA*	22°	22.0°	18.0°
*1-NA*	4 mm	7.0 mm	6.0 m
*1.NB*	25°	28.0°	18.0°
*1-NB*	4 mm	9.0 mm	5.0 m

*CoA*	83 mm	91.0 mm	92.1 mm
*CoGn*	100 mm	131.0 mm	131.1 mm
*LAFH*	57–59 mm	81.0 mm	82.5 mm
*Facial angle*	90° ± 3°	90.0°	90.0°
*A-Nperp*	0-1 mm	−0.9 mm	0 mm
*Pg-Nperp*	0 ± 2 mm	−3.0 mm	−3.0 mm
*H line*	9 a 11 mm	5.0 mm	9.0 mm
